# External validation of a prognostic model based on total tumor load of sentinel lymph node for early breast cancer patients

**DOI:** 10.1007/s10549-020-05623-4

**Published:** 2020-04-06

**Authors:** Antonio Piñero-Madrona, Francisco Ripoll-Orts, José Ignacio Sánchez-Méndez, Asunción Chaves-Benito, Maximiliano Rodrigo Gómez-de la Bárcena, Ana Calatrava-Fons, Salomón Menjón-Beltrán, Vicente Peg-Cámara

**Affiliations:** 1 Breast Cancer Unit, Department of Surgery, Virgen de La Arrixaca University Hospital, Ctra. Madrid-Cartagena, s/n, El Palmar, 30120 Murcia, Spain; 2 Breast Cancer Unit, La Fe University and Polytechnic Hospital, Valencia, Spain; 3 Breast Unit, La Paz University Hospital, Madrid, Spain; 4 Pathology Department, J.M. Morales Meseguer Hospital, Murcia, Spain; 5 Pathology Department, Burgos University Hospital, Burgos, Spain; 6 Pathology Department, Fundation Institute of Oncology, Valencia, Spain; 7 Oncologic Gynaecology Unit, Virgen de Las Nieves Hospital, Granada, Spain; 8 Pathology Department, Vall D’Hebron University Hospital, Barcelona, Spain

**Keywords:** Breast cancer, Prognosis, Sentinel lymph node, Total tumor load, Model validation

## Abstract

**Background:**

A prognostic model based on the results of molecular analysis of sentinel lymph nodes (SLN) is needed to replace the information that staging the entire axilla provided. The aim of the study is to conduct an external validation of a previously developed model for the prediction of 5-year DFS in a group of breast cancer patients that had undergone SLN biopsy assessed by the One Step Nucleic Acid Amplification (OSNA) method.

**Methods:**

We collected retrospective data of 889 patients with breast cancer, who had not received systemic treatment before surgery, and who underwent SLN biopsy and evaluation of all SLN by OSNA. The discrimination ability of the model was assessed by the area under the ROC curve (AUC ROC), and its calibration by comparing 5-years DFS Kaplan–Meier estimates in quartile groups of model predicted probabilities (MPP).

**Results:**

The AUC ROC ranged from 0.78 (at 2 years) to 0.73 (at 5 years) in the training set, and from 0.78 to 0.71, respectively, in the validation set. The MPP allowed to distinguish four groups of patients with heterogeneous DFS (log-rank test *p* < 0.0001). In the highest risk group, the HR were 6.04 [95% CI 2.70, 13.48] in the training set and 4.79 [2.310, 9.93] in the validation set.

**Conclusions:**

The model for the prediction of 5-year DFS was successfully validated using the most stringent form of validation, in centers different from those involved in the development of the model. The external validation of the model confirms its utility for the prediction of 5-year DFS and the usefulness of the TTL value as a prognostic variable.

**Electronic supplementary material:**

The online version of this article (10.1007/s10549-020-05623-4) contains supplementary material, which is available to authorized users.

## Introduction

The sentinel lymph nodes (SLN) biopsy is consolidated as an effective procedure in the staging of solid tumors, especially in breast cancer [1–3]. For years, the exhaustive study of these lymph nodes through immunohistochemical techniques, and particularly molecular methods [4] have resulted in an increased detection of minimal neoplastic involvement, which promoted research on its biologic and prognostic relevance in early diagnostics of breast cancer.

Several studies were conducted to determine the total tumor load (TTL) that would allow to avoid unnecessary axillary lymph node dissection (ALND), based on the prediction of non-SLN affectation [5–8]. The ACOSOG Z0011 study published in 2011 allowed to identify a group of breast cancer patients with positive SLN in which ALND could be omitted. This resulted in a considerable increase in the number of patients with affected SLN and no ALND [9–11].

Nodal involvement (pN) has been always considered one of the main prognostic predictors in early breast cancer [12]. However, due to the previously mentioned research works, its study is now restricted in many cases to the SLN (pN_(sn)_), which might compromise its prognostic value. The PLUTTO study [13] was developed as the next logical step to collect evidence of the prognostic value of the TTL, defined as the sum of the copy number of CK19 mRNA detected in every SLN examined, expressed as a concentration (copies/μL).This study concluded that the TTL allows to distinguish two groups of patients with different overall survival (OS) and disease-free survival (DFS), independently from other known prognostic factors, using a threshold of 25,000 copies/μL. From this evidence, prognostic models were developed for DFS, loco-regional DFS (LRDFS) and OS, based on TTL, the patient’s age, and a risk score derived from tumor characteristics.

The primary objective of the present study was to conduct an external validation of a previously developed model [13] for the prediction of 5-year DFS in patients with a diagnostic of breast cancer that had undergone SLN biopsy with TTL determination from one step nucleic amplification (OSNA) method. Secondary objectives were the validation of analogous models for LRDFS and OS.

## Patients and methods

We collected longitudinal data of a historic cohort of patients with breast cancer from eight different Spanish hospitals, who underwent SLN biopsy January 2008 to June 2014, and evaluation of all SLN by OSNA (without conventional histological evaluation). Exclusion criteria were carcinoma in situ, tumors with no expression of Cytokeratin 19 (CK19), cases with systemic primary treatments (chemotherapy or hormonotherapy), lack of information related to demographics, tumor, sentinel lymph node biopsy, breast cancer treatment and follow-up in the clinical records, or when OSNA evaluation of SLN was not made with all, complete, excised SLN. The study was approved by the Ethical Review Board of each institution (reference IRB: 2017-4-3-HCUVA, Hospital Clínico Universitario Virgen de la Arrixaca, Murcia, Spain). Given its retrospective nature, informed consent was not obtained, but patient privacy was protected by using a patient study number in the data collection and through a dissociated management of the data.

Data collected for each patient included clinicopathological characteristics as well as clinical follow-up. The risk score derived from tumor characteristics was computed by adding up the number of risk factors (size > 2 cm, lymphovascular invasion, ki67 > 20, HER2 positive, ER negative, PR negative, and tumor grading) [13]. Follow-up time was defined as the time from the SLN biopsy date to the last follow-up date or the event date (progression or death), whichever came first. Progression events were classified as local, regional or distant.

### Statistical analysis

The sample size was determined using estimates from a previous study [13]. At least 25 events per variable have been advised as a minimum for the external validation of prognostic models [14]. Since the model to be validated has three variables (age, TTL and a risk score), 75 events were needed. Because this was exactly the number of events in the previous study (*n* = 950 patients), we planned to recruit 1000 patients in the current study, allowing for a 5% of invalid cases.

Results are presented for both the cohort previously used to develop the prognostic models (training set), and the current study cohort (validation set). Data are described as counts (*n*) and percentages, mean (SD) or median (IQR), as appropriate.

According to recommendations for the validation of prognostic Cox regression models [15], we proceeded as follows: (1) the Prognostic Index (PI), defined as the linear predictor of the previous Cox model equation fitted in the training set, was computed for the validation and training sets; model predicted probabilities (MPP) were also computed, without recalibration in the training set; (2) the PI and MPP distributions in both the training and validation sets were compared by graphical methods (density plots); (3) we estimated the regression coefficient for the PI in the test set, and we tested the hypothesis of unit slope by the likelihood ratio test; (4) a possible model misspecification or lack of fit was assessed by fitting a model including the PI with unit slope, as well as the variables used in developing the model (age, TTL and risk score), and testing the hypothesis that the coefficients of these variables are all null; (5) the discriminant ability of the model was assessed by computing the area under the ROC curve (AUC ROC), and its calibration by comparing the Kaplan–Meier curves for groups defined by the quartiles of the MPP, in the training and validation sets. Hazard ratios (HR) for the three groups of higher risk were computed using a Cox model.

The log-rank test was used to compare survival curves. Statistical significance was declared in all analyses if *p* < 0.05. All analyses were conducted with the R language (version 3.5.0) [16].

## Results

A total of 1089 patients from eight sites were considered for inclusion in the validation study. However, 200 patients were excluded due to violation of selection criteria (4), lack of data required for model validation (193) or both (3), so that the validation set included 889 patients.

Table 1 shows the main characteristics of patients in the training and validation sets. In the 889 validation patients, the median follow-up was 6.4 years (Q1 5.6; Q3 7.0), 84 had a DFS event, 67 had a LRDFS event, and 51 died. A total of 1367 lymph nodes were analyzed, 544 (61.2%) patients had zero positive nodes, 278 (31.3%) one positive node, 55 (6.2%) had 2 positive nodes, 11 (1.2%) had 3 positives nodes and 1 (0.1%) had 4 metastatic SLNs. TTL values ranged from 0 to 27 × 10^6^ CK19 mRNA copies/μL, with a median of 0 and IQR 3100. In 544 cases, TTL was 0. In the remaining 345 patients who had non-null TTL values, the quartiles were 1400, 10^4^, and 10^5^. These figures were similar to those reported previously for the training set [13].

**Table 1:** Characteristics of patients in the training and validation sets

	Training *N* = 950	Validation *N* = 889
Age (years)*	58.4 (13.1)	57.5 (12.0)
Sex (female)	944 (99.4%)	886 (99.7%)
Tumor type		
Lobular	83 (8.7%)	67 (7.6%)
Ductal	797 (83.9%)	783 (88.2%)
Other	70 (7.4%)	38 (4.3%)
Tumor grade		
1	275 (28.9%)	226 (25.4%)
2	465 (48.9%)	424 (47.7%)
3	210 (22.1%)	239 (26.9%)
Tumor size (mm)**	16.0 [11.0; 22.0]	17.0 [12.0; 23.0]
Lymphovascular invasion	185 (19.5%)	231 (26.0%)
Estrogen receptors	837 (88.1%)	796 (89.5%)
Progesterone receptors	767 (80.7%)	720 (81.0%)
HER2 overexpression	108 (11.4%)	118 (13.3%)
ki67 (%)**	15.0 [10.0; 25.0]	15.0 [8.00; 30.0]
Risk Score**	3.00 [2.00; 4.00]	3.00 [2.00; 5.00]

Data are summarized as *n* (%), mean (SD)*, or median [IQR]**, as appropriate

### Validation of the DFS prognostic model

**Fig. 1:**
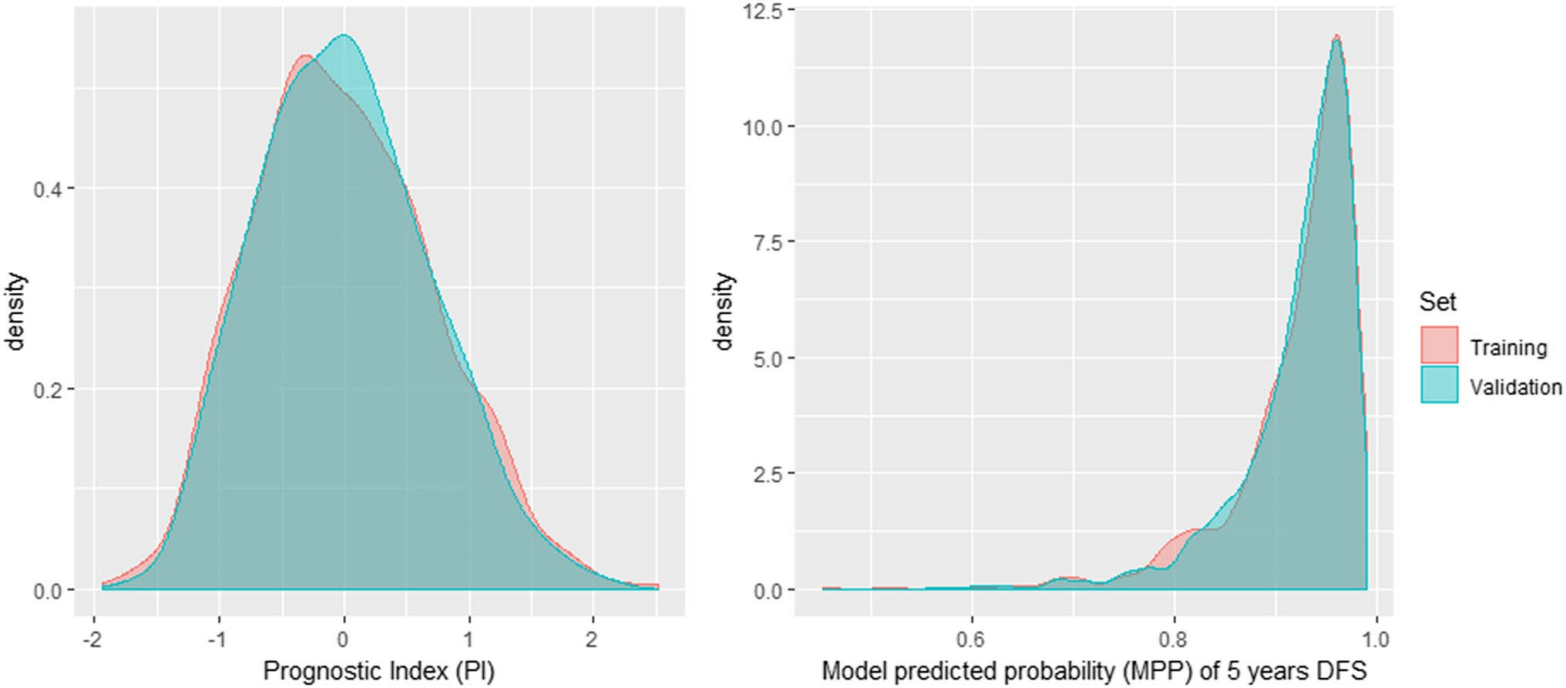
Distributions of the prognostic index (left) and model predicted probability of DFS at 5 years in the training and validation sets

Figure 1 shows the distribution of the PI and the MPP of 5-year DFS in the training and validation sets, that are almost identical.

When a Cox model was fitted to the validation data with the PI as single predictor, its slope did not significantly differ from 1 (*χ*^2^ = 0.031; d.f. = 1; *p* = 0.861) and was estimated as 1.03 [95% CI 0.72, 1.33]. Moreover, the test of a possible model misspecification by forcing the PI slope to be one and adding the age, the risk score and the log(TTL + 1) as predictors, did not result in a significant improvement of the fit (*χ*^2^ = 3.07; d.f. = 3; *p* = 0.38). The discrimination ability of the model, as measured by the AUC of the ROC curve, showed a slight decline over time (as it is usually the case for models based on predictors assessed at start of follow-up), ranging from 0.78 at 2 years, to 0.73 at 5 years in the training set, and from 0.78 to 0.71 in the validation set.

**Fig. 2:**
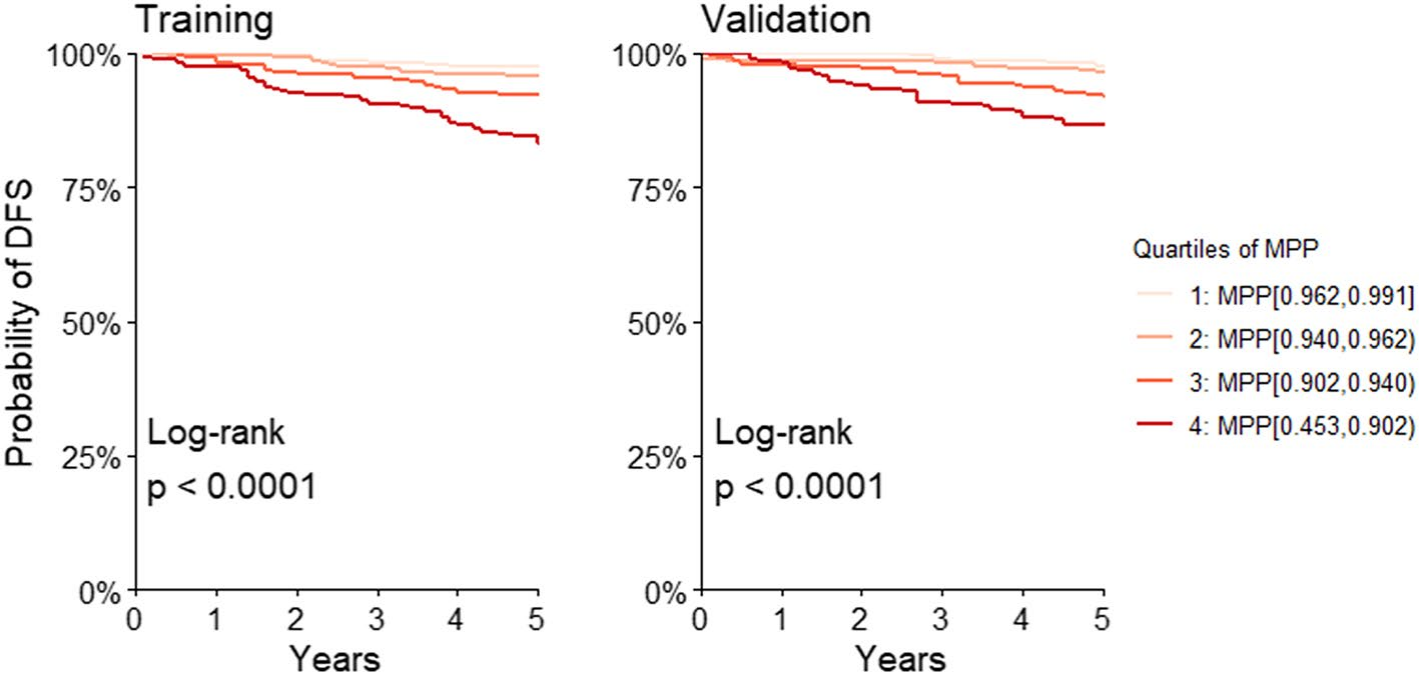
Kaplan–Meier curves of 5-year DFS in the four groups defined by quartiles of the model predicted probabilities (MPP), and log-rank test, in the training and validation sets

Figure 2 displays the Kaplan–Meier curves for 5-year DFS in the four groups defined by quartiles of the MPP. The four curves were heterogeneous in both the training and the validation set (log-rank test *p* < 0.0001). The curves are very similar in groups 1 and 2, but progressively worse for groups 3 and 4.

Table 2 documents the Kaplan–Meier estimates of 5-years DFS in the same four groups, and the hazard ratios (HR) for groups 2, 3 and 4 (group 1 as reference). The HR is not different from unit in group 2 but increases progressively in groups 3 and 4. The difference between 5-year DFS estimates in the lowest and highest risk groups was 0.975–0.831 = 0.144 (or 14.4%) in the training set, and 0.976–0.866 = 0.11 (or 11.0%) in the validation set.

**Table 2:** Kaplan–Meier estimates of 5-years DFS in groups defined by quartiles of MPP

Quartiles of MPP	Training (*n* = 950)		Validation (*n* = 889)	
	DFS (5 years)	HR [95% CI]	DFS (5 years)	HR [95% CI]
1: MPP [0.962, 0.991]	0.975	–	0.976	–
2: MPP [0.940, 0.962)	0.957	1.42 [0.54, 3.73]	0.963	1.00 [0.396, 2.53]
3: MPP [0.902, 0.940)	0.923	2.84 [1.19, 6.79]	0.918	2.86 [1.350, 6.06]
4: MPP [0.453, 0.902)	0.831	6.04 [2.70, 13.48]	0.866	4.79 [2.310, 9.93]

**Fig. 3:**
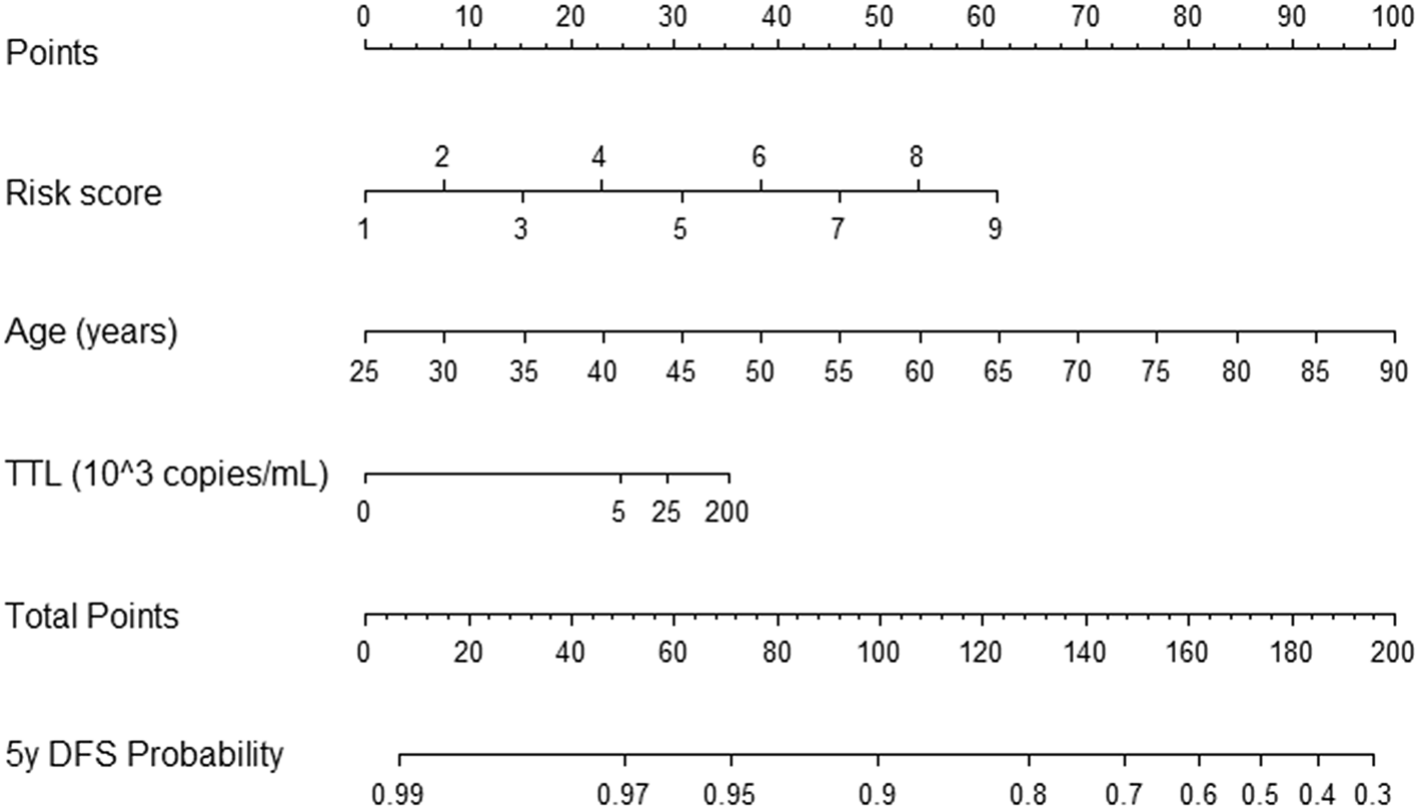
Nomogram of the validated model to predict probability of DFS at 5 years

A nomogram to facilitate the utilization of the model is provided in Fig. 3.

### Validation of the LRDFS and OS prognostic models

Prognostic models for LRDFS and OS that had been previously developed from the training data, were assessed using data from this validation study. In both cases, results were like those reported above for the DFS model, in that no evidence of model misspecification was found. For the LRDFS model, the discrimination ability measured by the AUCROC ranged from 0.78 (at year 2) to 0.74 (year 5) in the training set, and from 0.76 to 0.70, respectively, in the validation set. For the OS model, values were 0.78 (year 2) and 0.73 (year 5) in the training set, and 0.78 and 0.71, respectively, in the validation set.

### Prognostic value of TTL > 25,000 (copies/μL)

According to the TTL value (CK19 mRNA copies/μL), the 5-year DFS was lower in patients having TTL ≥ 25,000 than in those with TTL < 25,000 (*p* = 0.041).

## Discussion

Our results document the external validity of a model previously developed to predict DFS from age, a risk score based on tumor characteristics, and TTL, in early breast cancer patients in whom SLN analysis was conducted by molecular methods. This model performed well when applied to a new sample of patients (validation set) recruited from centers different from those involved in its construction (training set). The discrimination ability of the model (AUC ROC) ranged from 0.78 (at 2 years) to 0.73 (at 5 years) in the training set, and from 0.78 to 0.71, respectively, in the validation set. The quartiles of the MPP defined groups of patients with heterogeneous DFS curves (Fig. 2). The difference between the extreme groups in actual 5-years DFS probability was 14.4% in the training set, and 11.0% in the validation set (see Table 2 and text).

Our study also confirmed significant different 5-years DFS according to the 25,000 copies/μL TTL threshold. However, this was only done as a way of documenting that TTL may contribute valuable information for 5-years DFS, but we do not suggest the use of any threshold [17] for prediction or therapeutic decision making. In the model, TTL was introduced as a continuous variable, since categorization is unnecessary, and has been shown to be inefficient in prognostic model development [18].

Extensive work has been done on the prediction of non-SLN involvement from OSNA [5–8, 17, 19–22]. The prognosis of breast cancer in terms of DFS and overall survival, has been studied in clinical trials [2, 10], and observational studies proposing tools for prediction [3, 13, 23, 24], most of them basing the prediction on ALND and/or tumor markers.

The Nottingham Prognostic Index (NPI) was developed long ago, using tumor size, grade, and lymph node staging from 387 patients recruited in a single center [23]. Lymph node involvement was based on biopsy of “a lower axillary node, an apical axillary node and a node from the internal mammary chain”. The NPI was subsequently applied (using conventional axillary lymph node staging) to large samples of patients from other populations with a variable degree of success [25, 26], but these exercises did not follow the standard methodology for the validation of prognostic models, and measures of model discrimination were not reported. Recently, a newer version of the NPI (NPLI Plus) has been proposed to predict development of distant disease [3].

A recent study proposed a nomogram for the prediction of survival on the base of a modified lymph node ratio in breast cancer patients undergoing ALND [24]. In this study, patients that only received SLN biopsy were explicitly excluded (1328 out of 5736 patients that received surgery, or 23%), and therefore their model and ours should be considered complementary. Their model showed a good discrimination capacity (*c*-index 0.789 in the validation set), though this was assessed by split-sample rather than external validation.

Validating a prognostic model means establishing that it works satisfactorily for patients other than those from whose data the model was derived. Neither internal validation (data splitting or cross-validation) nor temporal evaluation (subsequent patients within the same centers) addresses the wider issue of the generalizability of the model. As the goal of validation is to demonstrate satisfactory performance for patients from a different population from the original, it is clearly desirable to evaluate a model on new data collected from an appropriate patient population in different centers. For this reason, external validation is considered to be the most stringent form of validation [15, 27].

As mentioned in the 8th edition of the American Joint Committee on Cancer (AJCC) for breast cancer, the biology of the tumor should be considered for clinical and prognostic information [28]. Axillary staging (pN) is still an independent prognostic factor in early breast cancer patients; however, after the publication of the ACOSOG Z0011 trial and others, the therapeutic significance of ALND became questionable limiting the prognostic information of axillary involvement only to sentinel lymph node (pN(sn). Several models show a good prognostic value for positive SNL nevertheless, our prognostic model for early breast cancer based in an objective factor as is TTL and other outcomes may improve the management and follow-up of breast cancer patients using this variable. The validated model helps to identify patients with a lower 5-years DFS.

Our study has several limitations. First, data were retrospectively collected and, in general, retrospective data are less accurate and more prone to bias than prospective data. However, because the data were prospectively recorded, and their nature is not subjective, we think there is little risk of important inaccuracies. High inclusion rate of estrogen receptor positive patients, 88.1% in the training set and 89.5% in the validation set, as a result of uniformity subtype group could be considered a prognostic bias, nevertheless, patients included are representing a real sample of the population. Concerning a potential risk of bias, given our selection criteria we cannot think of any mechanism that could introduce a distortion in the predictive ability of the model. Second, our model predicts DFS at 5 years, which is a minimum meaningful time to assess prognosis in early breast cancer. A longer time frame of 10 or 15 years would be of great interest, but the molecular analysis of SLN is a relatively novel method [4], and its wide adoption in our environment dates less than 10 year ago [6, 7]. Definitely, another limitation is the need to get OSNA method which is not available in all centers; however, the use of molecular methods is recommended for a better prognostic precision, provided that the center can have it [28].Last, despite our selection criteria were not very restrictive, not all breast cancer patients satisfy them.

## Conclusions

A prognostic model of 5-years DFS based on age, a tumor risk score, and TTL from SLN, was shown to be valid when applied to patients in centers different from those involved in its construction. The model was useful to identify patients that are at higher risk of disease progression, which may be interesting in a context of restrictive indication of ALND.

## Electronic supplementary material

Below is the link to the electronic supplementary material.

Supplementary file1 (DOC 93 kb)
