# Supplementary material for: External validation of a prognostic model based on total tumor load of sentinel lymph node for early breast cancer patients
Source: Breast Cancer Res Treat. 2020 Apr 6;181(2):339–45. doi: 10.1007/s10549-020-05623-4 (PMC7188708; doi:10.1007/s10549-020-05623-4)
Supplement: Supplementary file 1 — Supplementary file1 (DOC 93 kb) [file 10549_2020_5623_MOESM1_ESM.doc]

### Validation of the LRDFS prognostic model


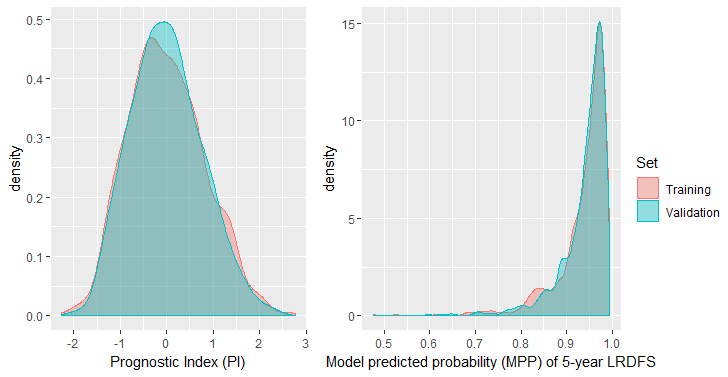


Figure 1. Distributions of the prognostic index (left) and model predicted probability of LRDFS at 5 years in the Training and Validation sets.


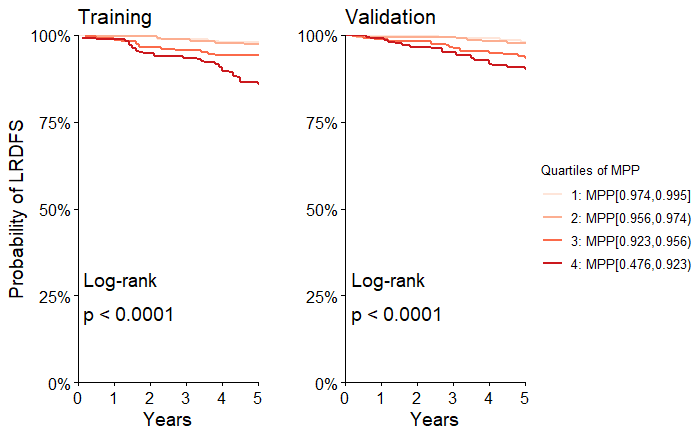


Figure 2. Kaplan-Meier curves of 5-year LRDFS in the four groups defined by quartiles of the model predicted probabilities (MPP), and log-rank test, in the Training and Validation sets.

Table 1. Kaplan-Meier estimates of 5-years LRDFS in groups defined by quartiles of MPP

|  | Training (n = 950) | | Validation (n = 889) | |
| --- | --- | --- | --- | --- |
| Quartiles of MPP | LRDFS (5y) | HR [95% CI] | LRDFS (5y) | HR [95% CI] |
| 1: MPP[0.974,0.995] | 0.979 | - | 0.981 | - |
| 2: MPP[0.956,0.974) | 0.973 | 1.45 [0.46, 4.56] | 0.977 | 1.11 [0.37, 3.32] |
| 3: MPP[0.923,0.956) | 0.942 | 3.39 [1.23, 9.34] | 0.935 | 3.07 [1.23, 7.64] |
| 4: MPP[0.476,0.923) | 0.859 | 7.62 [2.99, 19.42] | 0.902 | 6.24 [2.62, 14.87] |

### Validation of the OS prognostic model


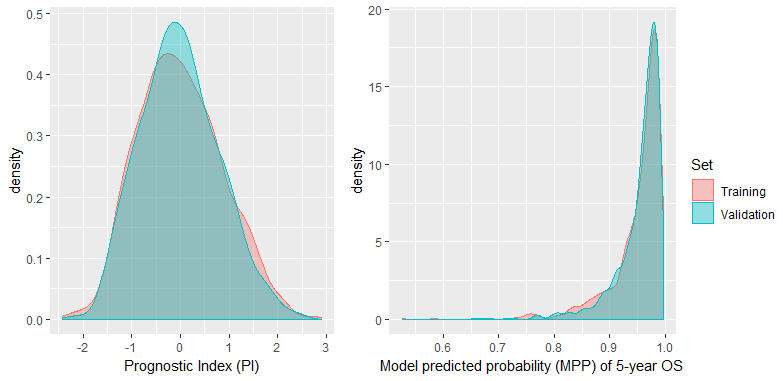


Figure 3. Distributions of the prognostic index (left) and model predicted probability of OS at 5 years in the Training and Validation sets.


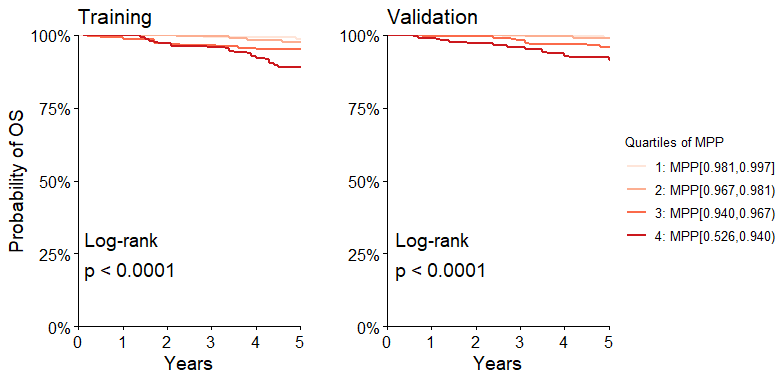


Figure 4. Kaplan-Meier curves of 5-year OS in the four groups defined by quartiles of the model predicted probabilities (MPP), and log-rank test, in the Training and Validation sets.

Table 2. Kaplan-Meier estimates of 5-years OS in groups defined by quartiles of MPP

|  | Training (n = 950) | | Validation (n = 889) | |
| --- | --- | --- | --- | --- |
| Quartiles of MPP | OS (5y) | HR [95% CI] | OS (5y) | HR [95% CI] |
| 1: MPP[0.981,0.997] | 0.986 | - | 0.990 | - |
| 2: MPP[0.967,0.981) | 0.977 | 2.11 [0.53, 8.42] | 0.991 | 1.40 [0.23, 8.39] |
| 3: MPP[0.940,0.967) | 0.952 | 4.39 [1.24, 15.54] | 0.960 | 7.09 [1.62, 31.00] |
| 4: MPP[0.526,0.940) | 0.889 | 10.24 [3.12, 33.62] | 0.912 | 17.06 [4.08, 71.31] |
